# 

**Published:** 1917-04

**Authors:** 


### ANNOUNCEMENTS

DEDICATION OF THE ROCHESTER DENTAL DISPENSARY, MAY 9, 1917. FOUNDED BY GEORGE EASTMAN. Rochester's new Dental Dispensary will be formally dedicated May 9 in conjunction with the Forty-ninth Annual Convention of the Dental Society of the State of New York, May 10, 11, 12. Men of national reputation will be here with messages of vital importance to you. Make your reservations today—The Committee. Edward G. Link, Chairman, 226 Cutler Bldg., Rochester, N. Y.; Louis Meisburger, Buffalo, N. Y.; Wirt H. Conklin, Rochester, N. Y.

---

THE INDIANA STATE DENTAL ASSOCIATION. The Fifty-Ninth Annual Meeting of the Indiana State Dental Association will be held at the Claypool Hotel in Indianapolis on May 15, 16, 17. A very good program of papers and clinics has been prepared and a sincere invitation is extended to members of other State Dental Associations to attend. A. R. Ross, Secretary.

---

AMERICAN INSTITUTE OF DENTAL TEACHERS. At the last annual meeting of the American Institute of Dental Teachers, held at Philadelphia, Pa., January 23 to 25, 1917, the following officers were elected: President, Dr. John F. Biddle, 517 Arch Street, Pittsburgh, Pa.; Vice-President, Dr. A. W. Thornton, McGill University, Montreal, Que.; Secretary-Treasurer, Dr. Abram Hoffman, 529 Franklin Street, Buffalo, N. Y.; Executive Board, Dr. R. W. Bunting, Ann Arbor, Mich.; Dr. A. D. Black, Chicago, Ill., and Dr. G. S. Millberry, San Francisco, Cal.

The next annual meeting will be held January 29, 30 and 31, 1918. The place of meeting will be announced later.

"IN MEMORIAM" RESOLUTIONS — DR. ISADORE LETT. WHEREAS, Our esteemed Fellow, Dr. Isadore Lett, has been removed from us by Divine Providence, it is fitting that we should make a record of his death and express our sorrow over the untimely close of his career; and

WHEREAS, By his genial disposition, his skill as a practitioner of dentistry, he has been a credit to the profession; therefore, be it

*Resolved,* That we, the Fellows of the American Academy of Dental Science, feeling deeply the loss we have sustained, hereby express our appreciation of his friendship, and our sorrow at his death; and be it further

*Resolved,* That these resolutions be spread upon our minutes, and that a copy be sent to the widow and his family.

CHARLES M. PROCTOR,
JEAN J. LOIZEAUX,
KNUT LUTTROPP.

---

TENNESSEE STATE MEETING. Don't forget that Tennessee celebrates its Golden Jubilee in Memphis June 19 to 22, and everybody will be welcomed.

---

KANSAS STATE BOARD OF EXAMINERS. The annual examinations for registration will be held at the National Hotel, in Topeka, on June 18. Application and fee of $25 must be in the hands of the secretary not later than June 8. Full particulars can be had by writing to Dr. C. F. Ambrose, El Dorado, Kan.

---

FOUR STATES' POST GRADUATE COURSE. Alabama, Mississippi, Texas and Louisiana will hold a four-day session in New Orleans, June 4 to 7, 1917. Specialists will give lectures and clinics that will be of great value and unusual interest. Drs. A. E. Smith and Carl G. Knocke, of Chicago; Dr. N. B. Nesbett, of Boston; Dr. Carl G. Lucas, of Indianapolis; Dr. J. P. Wall, of

New Orleans, are the special attractions. There will also be progressive clinics, moving picture exhibits and other interesting clinics, exhibits, etc. The headquarters will be at the Hotel Grunewald.

---

NORTHERN OHIO DENTAL ASSOCIATION. The next meeting of this society will be held in Cleveland, June 7 to 9, in the Hotel Statter. This is planned to be one of the best meetings of this old society.

---

KENTUCKY STATE SOCIETY. Meets May 9-11 in Louisville. W. M. Randall, Secretary, Louisville, Ky.

ILLINOIS STATE SOCIETY. Meets May 8-11 in Quincy, Ill. I. P. Luthringer, Secretary.

---

### INDIVIDUAL BASES FOR ARTIFICIAL TEETH

The late Sir Hiram Maxim had some very original ideas on artificial dentures and their need of improvement:

''You teeth-men are all wrong in your construction of artificial teeth,'' he said. ''Fancy constructing teeth all on the same base, and placing them on a tender place like the gums. Each tooth should be treated individually and independently; I am so busy just now with my inventions, otherwise I would soon construct some teeth and show you what I mean. Each artificial tooth should be imbedded in a cushion of air or soft rubber, so as to take up shock, and such shock should not be allowed to be imparted to a neighboring tooth. This is my idea of how an artificial denture should be constructed. Of course, it would have to be worked out properly, but it is on this principle that artificial dentures of the future will be constructed.''

It would seem an easy matter to employ flexible rubber in constructing a set as outlined, but it is another story to keep them in a sanitary condition with all the teeth on the denture and not in the wearer's stomach.—*Oral Hygiene.*

